# Transcriptome-wide identification and characterization of microRNAs in diverse phases of wood formation in *Populus trichocarpa*

**DOI:** 10.1093/g3journal/jkab195

**Published:** 2021-06-04

**Authors:** Ruiqi Wang, Mengxuan Reng, Shuanghui Tian, Cong Liu, He Cheng, Yingying Liu, Huaxin Zhang, Muhammad Saqib, Hairong Wei, Zhigang Wei

**Affiliations:** 1 State Key Laboratory of Tree Genetics and Breeding, Northeast Forestry University, Heilongjiang Harbin 150040, China; 2 Research Center of Saline and Alkali Land of State Forestry and Grassland Administration, Chinese Academy of Forestry, Beijing 100091, China; 3 Institute of Soil and Environmental Sciences, University of Agriculture, Faisalabad 38000, Pakistan; 4 College of Forest Resource and Environmental Science, Michigan Technological University, Houghton MI49931, USA

**Keywords:** miRNA, target gene, primary stems, transition stems, secondary stems, *Populus trichocarpa*

## Abstract

We applied miRNA expression profiling method to *Populus trichocarpa* stems of the three developmental stages, primary stem (PS), transitional stem (TS), and secondary stem (SS), to investigate miRNA species and their regulation on lignocellulosic synthesis and related processes. We obtained 892, 872, and 882 known miRNAs and 1727, 1723, and 1597 novel miRNAs, from PS, TS, and SS, respectively. Comparisons of these miRNA species among different developmental stages led to the identification of 114, 306, and 152 differentially expressed miRNAs (DE-miRNAs), which had 921, 2639, and 2042 candidate target genes (CTGs) in the three respective stages of the same order. Correlation analysis revealed 47, 439, and 71 DE-miRNA-CTG pairs of high negative correlation in PS, TS, and SS, respectively. Through biological process analysis, we finally identified 34, 6, and 76 miRNA-CTG pairs from PS, TS, and SS, respectively, and the miRNA target genes in these pairs regulate or participate lignocellulosic biosynthesis-related biological processes: cell division and differentiation, cell wall modification, secondary cell wall biosynthesis, lignification, and programmed cell death processes. This is the first report on an integrated analysis of genome-wide mRNA and miRNA profilings during multiple phases of poplar stem development. Our analysis results imply that individual miRNAs modulate secondary growth and lignocellulosic biosynthesis through regulating transcription factors and lignocellulosic biosynthetic pathway genes, resulting in more dynamic promotion, suppression, or regulatory circuits. This study advanced our understanding of many individual miRNAs and their essential, diversified roles in the dynamic regulation of secondary growth in woody tree species.

## Introduction

Wood represents the main source of terrestrial biomass production and is a major renewable resource for the timber, paper, and bioenergy industries (Zhong *et al.* 2010). Wood formation is a complexly continuous biological process involved in multiple molecular mechanisms to precisely control the collaborative expressions of many genes related to different processes of wood formation (Zhong *et al.* 2010, 2011; Quan *et al.* 2018). For example, a hierarchical transcriptional regulation network dominates secondary cell wall (SCW) formation, which mainly comprises the NAC and MYB transcription factor (TF) families and their regulated downstream structural genes related to SCW component biosynthesis and programmed cell death (PCD) (Zhong *et al.* 2011; Takata *et al.* 2019). Alternative splicing has been reported to occur within some genes related to SCW formation, such as *CESA* and *CCoMT* (Xu *et al.* 2014). Moreover, circRNAs were found to play essential roles in regulating genes involved in wood formation via circRNA-miRNA-mRNA networks (Liu *et al.* 2020). Until now, although extensive efforts have been made to unravel the molecular regulatory mechanisms of wood formation, a genome-wide profiling of both miRNAs and mRNAs across multiple developmental stages of wood formation may shed more light on the underlying regulatory mechanisms.

MicroRNAs (miRNAs), a group of 20- to 24-nucleotide (nt) small noncoding RNAs, are sequence-specific regulators that function mainly via post-transcriptional mRNA cleavage or the inhibition of gene expression in eukaryotes (Quan *et al.* 2016). More than 38,589 miRNAs have been identified (Release 22.1, October 2018; http://www.mirbase.org/). The available evidence indicates that miRNAs play influential roles in plant growth and development (Puzey *et al.* 2012; Quan *et al.* 2016). For example, miR393 participates in primary growth by repressing *TIR1/AFBs*, auxin receptor F-box genes (Yamamuro *et al.* 2016). csn-miR319c regulates apical bud burst by suppressing *CsnTCP2* in tea (Liu *et al.* 2019). miR528 promotes rice flowering by repressing *OsRFI2* under long-day conditions (Yang *et al.* 2019). Presently, although some miRNAs, such as miR875 (Zhao *et al.* 2015), ptr-miR397a (Lu *et al.* 2013), amg-miR166 (Chen *et al.* 2018), Pto-miR257 (Chen *et al.* 2016), Pto-MIR475b (Xiao *et al.* 2017), and miRNA156 (Wang *et al.* 2011b), have been identified to participate in different processes of wood formation (Du *et al.* 2011; Chen *et al.* 2018), no investigation has been reported on the dynamics of miRNAs and their biological functions involved in diverse phases of wood formation in tree species.

Presently, small RNA(sRNA) deep sequencing has emerged as a useful tool for plant miRNA rapid discovery, in which both the conserved and novel and lowly expressed miRNAs can be identified, and their abundances profiled simultaneously (Srivastava *et al.* 2015). To date, hundreds of sRNAs have been successfully isolated from model plants and nonmodel plants using this method (Srivastava *et al.* 2015). Moreover, because the expression levels of miRNAs display negative correlations with the expression levels of their target genes owing to transcript cleavage or translation repression (Rhoades *et al.* 2002; Brodersen *et al.* 2008), the biological functions of miRNAs can be effectively identified by integrated analysis of the miRNA and mRNA expression profiles (Rhoades *et al.* 2002; He *et al.* 2013). For example, through integrated analysis of the miRNA and mRNA profiles, some miRNAs have been identified to regulate the rapid growth of developing culms in *moso bamboo* (He *et al.* 2013), five miRNAs were found to be involved in ethylene-regulated petal growth in *Rosa hybrida* (Pei *et al.* 2013), and miR156a, miR157a, and miR172a have been validated to play important roles in the tuberous root development of *Brassica rapa* (Li *et al.* 2015).

The stems of less than 1-year-old poplar trees mainly comprise three developmental stages that include most of the processes of wood formation (Prassinos *et al.* 2005). For example, the segments near the apical meristems, where cells mainly undergo division and expansion and synthesis of the primary cell wall, are the primary stems (PSs) and represent the beginning phase of wood formation. By contrast, the segments in the basal portions, where cells undergo SCW biosynthesis, lignification, and PCD, are secondary stems (SSs) and represent the late phase of wood formation. In addition, segments in the middle of PS and SS, where cells synthesize SCW components in the inner part of the primary wall, are transition stems (TS) and represent the middle phase of wood formation. Thus, the different vertical segments of less than 1-year-old poplar trees have been used to investigate the dynamics of gene expression and molecular regulatory mechanisms underlying diverse phases of wood formation (Prassinos *et al.* 2005; Dharmawardhana *et al.* 2010; Liu *et al.* 2015a; Zhang *et al.* 2020). Based on the previous research of Zhang *et al.* (2020), in this study, we concurrently generated the miRNA and mRNA expression profiles in the PS, TS, and SS of *Populus trichocarpa* using Solexa sequencing. This study was designed to collect data and gain insight into three problems: (i) the dynamics of miRNAs associated with diverse phases of wood formation; (ii) the identification of the miRNA-mRNA model associated with diverse phases of wood formation; and (iii) the authentication of the biological functions of miRNAs related to diverse phases of wood formation. The sRNA-seq and RNA-seq data acquired constituted valuable genetic resources, and the results would be helpful for further studies of miRNAs involved in diverse processes of wood formation.

## Materials and methods

### Plant material

A few plantlets of *P. trichocarpa* clone Nisqually-1, whose genome was sequenced early, were obtained from the Center for Excellence in Molecular Plant Science, Chinese Academy of Sciences, and vegetatively propagated in our lab using tissue culture (Li *et al.* 2017). The plantlets were planted in humus soil and grown under 16/8 hours day/night photoperiod at 25°C in the greenhouse at Northeast Forestry University for 90 days. Then, Wang and Zhang *et al.* prepared 63 trees as experimental materials for transcriptome and sRNA sequencing, DNA methylation sequencing, qRT-PCR analysis, and anatomical and histological analysis, 9 average trees were selected as material for sequencing among of 63 trees (Zhang *et al.* 2020). According to the study of Prassinos *et al.* (2005) and Dharmawardhana *et al.* (2010), the IN2, IN4, and IN8 internodes were PS, TS, and SS stages from primary growth to secondary growth of poplar and represent the beginning, middle, and late phases of wood formation, respectively. Therefore, the IN2, IN4, and IN8 internodes were harvested for sample preparation from the stem of 9 trees, each sample was prepared by mixing the 3 same position internodes. These materials were sampled and immediately frozen in liquid nitrogen and stored at −80°C. The stem samples used in this study were the same as those used in Zhang’s study (Zhang *et al.* 2020).

### RNA extraction, library construction, and sRNA sequencing

Total RNA was extracted using Trizol (Invitrogen) according to the manufacturer’s protocol, and the quantity and integrity of RNA were examined using a 2100 Bioanalyzer with the RNA 6000 Nano Kit (Agilent Technologies, Santa Clara, CA, USA). Sequencing libraries were generated using NEBNext^®^ Multiplex sRNA Library Prep Set for Illumina^®^ (NEB, USA.) following manufacturer’s recommendations. Each sample build a library, all the nine libraries were for sRNA sequencing using Illumina Solexa Genome Analyzer II was according to the manufacturer’s protocol (Illumina, San Diego, CA, USA).

### Transcriptome sequencing and gene expression analysis

The same plant materials used for sRNA sequencing were also for transcriptome sequencing. The materials were first ground to powder in liquid nitrogen and total RNA was isolated using an RNA isolation kit (Auto Lab Biotechnology, Beijing, China). Using the RNase-Free DNase Set (Qiagen), we performed on-column DNase digestions three times during the RNA purification. The quantity and quality of extracted RNA were determined with a NanoDrop ND-1000 (Thermo, USA).

Whole transcriptome libraries were constructed using the NEB Next Ultra Directional RNA Library Prep Kit for Illumina (NEB, Ipswich, MA, USA) according to the manufacturer’s instructions; the resulting libraries were assessed for size, quantitation, integrity, and purity using a Bioanalyzer 2100 system and qPCR (Kapa Biosystems, Woburn, MA, USA). The libraries with good quality were subsequently sequenced on a HiSeq 2500 instrument that was set to produce 125 bp paired-end reads of 125 nucleotides long. Raw sequences were cleaned as follows: (1) remove reads containing adapters (a. remove adapters and the parts after adapters; b. discard the reads if they were less than 50 nt in length after removing adapter; otherwise, keep them.); (2) remove reads containing more than 10% unknown nucleotides (N); (3) remove low quality reads containing more than 50% low-quality bases (*Q*-value ≤ 20). After that, the clean reads from all the samples were mapped to the *P. trichocarpa* genome using TopHat2 software with default parameters. The expression levels of the protein-coding genes were calculated and normalized using fragments per kilobase of gene per million mapped fragments (FPKM) by Cufflinks (version 2.2.1).

### Analysis of sRNA sequencing data

From the raw sequence reads obtained from the sRNA sequencing, we first removed low-quality reads containing more than one low-quality (*Q*-value ≤ 20) base or containing unknown nucleotides(N). Then, removing reads with a 3’ adapter only, 5’adapter only (according to the known inherent sequence of 5’adapter contaminants), 3’ and 5’ adapters but without sRNA fragment between them, ployA in sRNA fragment, and shorter than 18 nt (not include adapters). Then clean reads were obtained without any mismatches.

### sRNA reads annotation and miRNA identification

All clean reads were annotated as follows. First, the sRNAs were aligned with the GenBank (http://www.ncbi.nlm.nih.gov/genbank/) and Rfam (11.0 release, http://rfam.xfam.org/) databases using task blastn-short of blast+(https://blast.ncbi.nlm.nih.gov/), the rRNA, scRNA, snoRNA, snRNA, and tRNA were identified and removed. Subsequently, all of the clean reads were also aligned with *P. trichocarpa* genome (https://phytozome-next.jgi.doe.gov/info/ Ptrichocarpa_v3_0) using BOWTIE (http://bowtie-bio.sourceforge.net/). Those reads that were mapped to exons or introns might be fragments from mRNA degradation, and were removed. The Repeat Masker was used to remove the repeat-associated RNAs (http://www.girinst.org/). Finally, the remaining clean reads alignment to miRNA sequence of miRBase (Release 22, http://www.mirbase.org) by using BOWTIE to identify known miRNAs. A sRNA that had a perfect match with a *P. trichocarpa* miRNA sequence was considered to be an existing miRNA, and a sRNA that did not match any *P. trichocarpa* miRNA sequence but a miRNA sequence in other species was considered to be a conserved miRNA. The nomenclature rules for conserved miRNAs is: *x* and *y* indicate that miRNAs are processed from the 5’ and 3’ arms of the precursor, respectively. For example, miR165-x and miR165-y. The existing miRNAs use 5p, 3p to indicate that miRNAs are processed from the 5’ and 3’ arms of the precursor, respectively. For example, ptc-miR6425b-5p and ptc-miR6425b-3p.

After identifying all existing and conserved miRNA, the remaining unannotated sRNA tags were used to predict novel miRNAs. MIREAP (https://sourceforge.net/projects/mireap/) was also used to predict potential the novel miRNAs by exploring their secondary structures, the dicer cleavage sites and the minimum free energy of the unannotated sRNA tags, the specific parameters coded in this software include (1) the minimal miRNA sequence length was 18 nt; (2) the maximal miRNA sequence length is 25 nt; (3) the minimal miRNA reference sequence length was 20 nt; (4) the maximal miRNA reference sequence length was 23 nt; (5) the maximal copy number of miRNAs on reference is 20; (6) the maximal free energy allowed for a miRNA precursor was 18 kcal/mol; (7) the maximal space between miRNA and miRNA* is 300 nt; (8) the minimal space between miRNA and miRNA* was 16 nt; (9) the maximal bulge between miRNA and miRNA* was 4 nt; (10) the maximal asymmetry of miRNA/miRNA* duplex was 4 nt; (11) the flank sequence length of miRNA precursor was 20 nt; the secondary structures of miRNA precursor were predicted by MFOLD (http://www.unafold.org/mfold/applications/rna-folding-form.php) using the default parameters (Zuker 2003). When a perfect stem-loop structure formed, the sRNA sequence should be located at one arm of the stem alone. In this case, this sRNA is considered to be a novel miRNA.

### Identification of DE-miRNAs and DEGs

Differentially expressed miRNAs (DE-miRNAs) among PS, TS, and SS were identified with edgeR package using the raw counts. The miRNA frequency from the three types of libraries was normalized to get the abundance of transcript per million (TPM): normalized miRNA expression = (actual miRNA count/total count of clean reads) × 1,000,000. The abundance changes among PS, TS, and SS were calculated as log2 (TS/PS, SS/PS, or SS/TS). miRNAs with an absolute |log2(FC)|≥1 and a *P*-value ≤ 0.05 were thought to be changed significantly and were considered as DE-miRNA.

Differentially expressed genes (DEGs) among PS, TS, and SS were also identified with edgeR package using the FPKM value. Genes exhibiting difference of |log2(FC)|≥1 with FDR ≤ 0.05 were considered as differentially expressed.

### miRNA target gene prediction

The candidate target genes (CTGs) of DE-miRNAs were predicted by using stand-alone version PatMatch software (Version 1.2) (Yan *et al.* 2005). We used the gene sequences identified by transcriptome sequencing to build a target dataset, DE-miRNAs match the genes in target dataset abiding by rigorous parameters as follows: (1) No more than four mismatches between sRNA/target (G-U bases count as 0.5 mismatches); (2) No more than two adjacent mismatches in the miRNA/target duplex; (3) No adjacent mismatches in positions 2–12 of the miRNA/target duplex (5’ of miRNA); (4) No mismatches in position 10–11 of miRNA/target duplex; (5) No more than 2.5 mismatches in position 1–12 of the miRNA/target duplex (5’ of miRNA); and (6) Minimum free energy (MFE) of the miRNA/target duplex should be ≥74% of the MFE of the miRNA bound to it’s perfect complement.

### Correlation analysis between the DE-miRNAs and their CTGs

Analysis to identify correlations between DE-miRNA and CTG expression in PS *vs* TS, PS *vs* SS, and TS *vs* SS comparison was done using the Pearson method. Then for each DE-miRNA and its predicted CTGs, Pearson correlation coefficient was calculated, and a contingency Table was created for all CTGs, which was used to assess the level of the negative correlated CTGs (−1 <correlation coefficient< −0.8 and *P*-value ≤ 0.05 was considered as a negative correlation) within predicted CTGs of the intended DE-miRNA.

### GO enrichment and KEGG pathway analysis

To further elucidate the potential regulatory roles of DE-miRNAs, we performed GO enrichment analysis of CTGs that are targets of DE-miRNAs. We first annotated CTGs using Blast2GO (version 5.2) and the Nr database (r20200419) (Conesa *et al.* 2005). GO enrichment was performed using the Pop’s Pipes (Li *et al.* 2014), a free online platform for data analysis (http://sys.bio.mtu.edu/), and the GO enrichment results were expressed as three independent hierarchies for molecular function, biological process, and cellular component. Putative CTGs related to metabolism in the cellular processes group were analyzed and annotated according to the Kyoto Encyclopedia of Genes and Genomes (KEGG) database (http://www.genome.jp/kegg/kegg1.html). The KEGG pathway annotation was accomplished using Blastp (https://blast.ncbi.nlm.nih.gov/) program against the KEGG database according to the method described by Kanehisa et al (Kanehisa *et al.* 2008). Pathway and GO enrichment were performed using the GO and KEGG databases with FDR/*P*-adjust ≤ 0.05 as a threshold.

The DEGs resulting from PS *vs* TS, PS *vs* SS, and TS *vs* SS comparisons were also subjected to GO enrichment and KEGG analyses.

### Real-time quantitative PCR analysis of miRNAs and CTGs

Total RNA was isolated from the same materials of PS, TS, and SS used to sRNA and expression profile sequencing with RNAiso plus (Takara, China) according to the manufacturer’s instructions. The first cDNA strand was synthesized from total RNA using the TransScript miRNA-reverse transcriptase (RTase) (Trans, China) and miRNA specific stem-loop primers were designed according to the method described by Chen *et al.*(Chen *et al.* 2005). Briefly, six nucleotides that paired with the 3’ end of the miRNA were linked to a stem-looped sequence (GTCGTATCCAGTGCAGGGTCCGAG GTATTCGCACTGGATACGAC) to synthesize the stem-loop reverse transcription primer. For target genes, the first cDNA strand was synthesized from total RNA using the TransScript RT/RI RTase (Trans, China). The next steps were identical to the reverse transcription of miRNA.

The expression change levels of miRNAs or CTGs were assessed on an ABI 7500 Fast Real-Time PCR instrument (Applied Biosystems, USA) with TransStart Top Green qPCR SuperMix (Trans, China), and the 5.8S rRNA gene was used as the endogenous reference. For target genes, the *β-actin* was selected as reference gene for normalization. 2^−ΔΔCT^ relative quantification method was used to analyze the relative changes of miRNAs and their targets expression in qPCR experiments (Livak and Schmittgen 2001). All of the qPCR reactions were conducted in three replicates. Standard errors and standard deviations were calculated from replicates.

### Data analysis

Standard errors and standard deviations were calculated by using SPSS 21 (Chicago, IL, USA). A statistically significant level was set to a *P*-value ≤ 0.05. The data are presented as mean ± standard error (SE) with each SE being calculated from three independents biological samples.

### Data availability

The datasets used and analyzed in this study are available in Sequence Read Archive (SRA) at NCBI at National Center for Biotechnology Information. The accession numbers for miRNA-seq data and RNA-seq data are PRJNA690170 and PRJNA628501, respectively. The datasets can be accessed at SRA (https://www.ncbi.nlm.nih.gov/sra) with the accession number provided above or https://www.ncbi.nlm.nih.gov/bioproject/PRJNA690170 and https://www.ncbi.nlm.nih.gov/bioproject/PRJNA628501.

Supplementary material is available at figshare: https://doi.org/10.25387/g3.14714880.

## Results

### sRNAs in diverse developmental stages of *P. trichocarpa* stems

To identify the miRNAs involved in diverse phases of wood formation, we performed high-throughput sequencing of sRNA libraries generated from PS, TS, and SS, and obtained 15,374,878, 15,653,777, and 11,989,204 high-quality reads, respectively. Subsequently, the adapter sequences and their artifacts (3’adapters only, 5’adapters only, 3’ and 5’ adapters without sRNA fragments between them), poly A sequences and the sequences <18-nt were removed, obtaining 14,705,980, 14,516,320, and 10,955,984 clean reads from PS, TS, and SS, respectively (Supplementary Table S1). After the further removal of rRNAs, tRNAs, snRNAs, snoRNAs, repeat-associated sRNAs, and exon and intron sequences, we finally obtained 12,237,495, 11,639,629, and 9,336,272 sRNA reads in PS, TS, and SS, respectively (Table 1). Among these sRNAs, nonannotated sRNAs accounted for the greatest proportion of sRNAs, about 31.17, 32.12, and 23.18% of the total tag abundances in PS, TS, and SS, respectively (Table 1). These sRNAs were used to predict and identify novel miRNAs implicated in wood formation of poplar.

**Table 1:** Distribution of tags among different categories in PS, TS, and SS of *P. trichocarpa*

Sample	PS	%	TS	%	SS	%
Total	14,705,980	100.00	14,516,320	100.00	10,955,984	100.00
rRNA	573,687	3.90	883,279	6.08	544,309	4.97
snRNA	14,216	0.10	16,238	0.11	9851	0.09
snoRNA	33,487	0.23	26,283	0.18	10,808	0.10
tRNA	64,517	0.44	93,329	0.64	51,681	0.47
Exon sense	624,595	4.25	723,074	4.98	431,574	3.94
Exon antisense	309,513	2.10	269,977	1.86	140,055	1.28
Intron sense	584,685	3.98	584,392	4.03	276,301	2.52
Intron antisense	208,812	1.42	210,260	1.45	109,456	1.00
Repeat	54,974	0.37	69,860	0.48	45,677	0.42
miRNA	7,653,018	52.04	6,977,646	48.07	6,796,138	62.03
nonannotated sRNA	4,584,477	31.17	4,661,983	32.12	2,540,134	23.18

**Figure 1:**
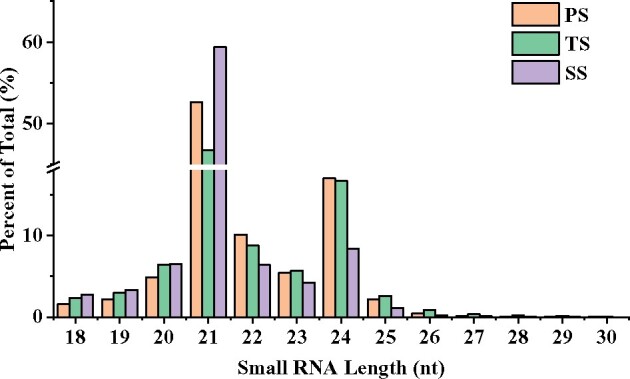
Length distribution of 18–30 nt sRNAs in PS, TS, and SS libraries. The *x*-axis represents different lengths of sRNA, and the *y*-axis represents percentage of sRNA in total. PS, TS, and SS represent primary stage, transition stage, and secondary stage of stem of *Populus trichocarpa*, respectively.

Most of the total sRNA reads of PS, TS, and SS ranged from 18- to 24-nt in lengths (Figure 1), slightly different than the typical size range (20–24 nt) for Dicer-derived products (Zhang *et al.* 2009a), and the 21- and 24-nt sRNA accounted for most of total RNA sequencing reads and represented the plurality of sRNAs. The abundance of 21-nt sRNAs was higher than that of sRNAs of any other lengths, and 24-nt sRNA represented the second highest abundant sRNA (Figure 1). The 21-nt sRNA accounted for 57.41, 51.12, and 45.28% of the total RNA reads in SS, PS, and TS, respectively, whereas the 24-nt sRNA accounted for 8.08, 16.48, and 16.13% of the total RNA reads in SS, PS, and TS, respectively (Supplementary Figure S1). The results indicate that the proportions of 21-nt and 24-nt sRNA in PS, TS, and SS of poplar were different. Based on Duncan’s multiple range test, the 21-nt sRNA in SS is significantly higher than those in PS and TS, the 24-nt sRNA in SS is significantly lower than those in PS and TS (Supplementary Figure S1).

### Known and novel miRNAs in the diverse developmental stages of *P. trichocarpa* stems

**Figure 2:**
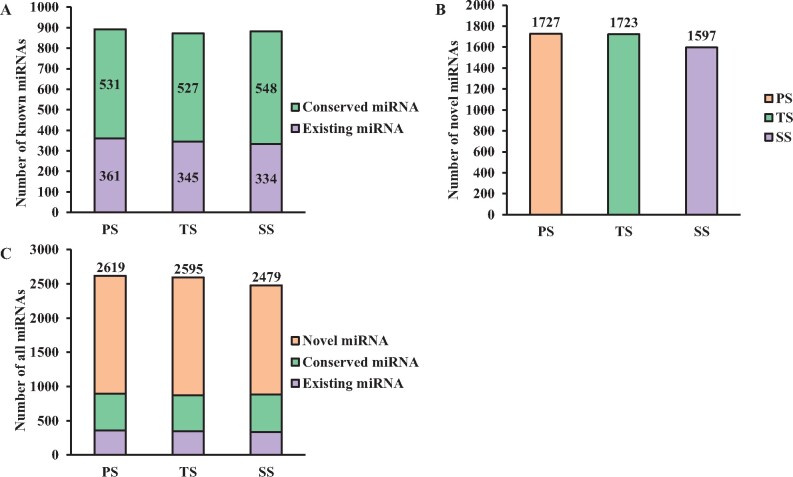
The number of different miRNAs in PS, TS, and SS of poplar. (A) The number of known miRNAs miRNA in different development stage. (B) The number of novel miRNAs in different development stage. (C) All identified miRNAs in different development stage.

In this study, only the miRNAs whose precursors can form hairpin structures were considered. According to this criterion, we aligned all sRNAs identified from PS, TS, and SS with miRNA sequences presented in miRBase22.1 of *P. trichocarpa.* Those sRNAs that could not be annotated to any known miRNAs were then used to identify potential novel miRNAs using MIREAP software if their precursors could form hairpin structures (Supplementary Figure S2). Consequently, 1310 known miRNAs were detected in PS, TS, and SS, containing 371 existing miRNAs and 939 conserved miRNAs (Supplementary Table S2). The numbers of the existing miRNAs and conserved miRNAs exhibited differences among PS, TS, and SS, which had 361, 345, and 334 existing miRNAs and 531, 527, and 548 conserved miRNAs, respectively (Figure 2A). Moreover, we identified 1727, 1723, and 1597 novel miRNAs with high reliability from nonannotated sRNAs of PS, TS, and SS, respectively (Supplementary Table S3 and Figure 2B). In summary, we identified 2619, 2595, and 2479 miRNAs from PS, TS, and SS containing 361, 345, and 334 existing miRNAs, 531, 527, and 548 conserved miRNAs, 1727, 1723, and 1597 novel miRNAs, respectively (Figure 2C).

In addition, we observed that the abundances of the above-identified miRNAs displayed differences among PS, TS, and SS (Supplementary Table S2). For example, the miRNA family with the highest abundance in PS and TS was the ptc-miR166 family, which had a TPM of 21,349,509 in PS and 8,730,386 in TS. The ptc-miR319 family, with 709,709 TPM, was the second-highest abundant miRNA family in PS, while the ptc-miR396 family, with 286,019 TPM, was the second-highest abundant miRNA family in TS. Finally, the ptc-miR396, with a TPM of 5,034,301, was the highest abundant miRNA species while the ptc-miR319, with a TPM of 592,466, was the second-highest abundant miRNA species in SS. Moreover, the miRNAs from the same family had distinct expression abundances among PS, TS, and SS (Supplementary Table S2). For example, the abundances of ptc-miR396a and ptc-miR396b increased from PS to SS, but ptc-miR396c, ptc-miR396d, and ptc-miR396e-5p showed higher abundances in TS than in PS and SS. These results demonstrate that the miRNAs of the same family or different families had different expression patterns among different developmental stems of poplar, suggesting that they could play various roles in diverse phases of wood formation.

### Differentially expressed miRNAs and their candidate target genes in diverse developmental stages of *P. trichocarpa* stems

**Figure 3:**
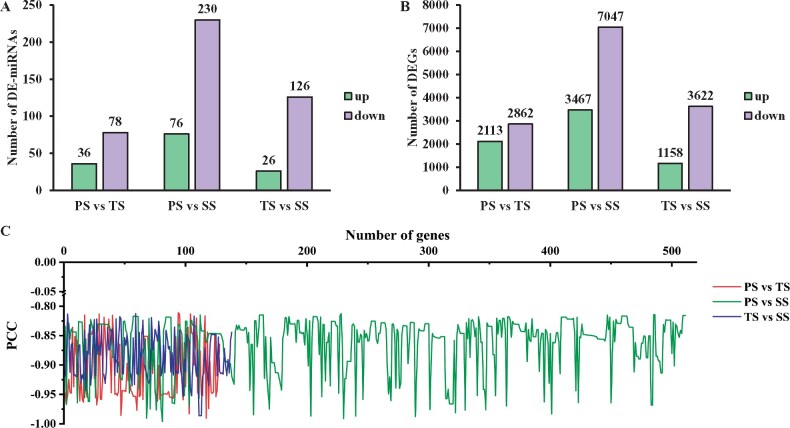
(A) differentially expressed (DE)-miRNA quantity statistics. (B) DEG quantity statistics. (C) Distribution of significant negative correlations of miRNA-target pairs. The *x*-axis of (c) represents the number of miRNA-target pairs, and the *y*-axis represents the Pearson correlation coefficients (PCCs). PS, TS, and SS represent primary stage, transition stage, and secondary stage of stem of poplar, respectively.

A comparative analysis was performed to identify the differentially expressed miRNAs (DE-miRNAs) involved in diverse developmental phases of wood formation. 114 (36 up-regulated and 78 down-regulated), 306 (76 up-regulated and 230 down-regulated), and 152 (26 up-regulated and 126 down-regulated) DE-miRNAs were identified in PS *vs* TS, PS *vs* SS, and TS *vs* SS, respectively (Figure 3A and Supplementary Tables S4–S6).

To identify the target genes of DE-miRNAs by integrated analysis of the miRNA and mRNA expression profiles, we concurrently performed mRNA-seq using the same materials as those for sRNA-seq. By comparing the expression levels of genes among PS, TS, and SS, we obtained 4975 (2113 up-regulated and 2862 down-regulated), 10,514 (3467 up-regulated and 7047 down-regulated), and 4780 (1158 up-regulated and 3622 down-regulated) DEGs in PS *vs* TS, PS *vs* SS, and TS *vs* SS, respectively (Figure 3B and Supplementary Tables S7–S9). To determine the main biological functions of the DEGs, Gene Ontology (GO) and Kyoto Encyclopaedia of Genes and Genomes (KEGG) were performed to annotate DEGs using Blast2GO and BLASTP program, respectively, we found that many of these DEGs were related to different processes and pathways of wood formation (Supplementary Tables S10–S15).

We predicted 921, 2639, and 2042 CTGs for 114, 306, and 152 corresponding DE-miRNAs using PatMatch software (Version 1.2) in PS *vs* TS, PS *vs* SS, and TS *vs* SS, respectively. However, these CTGs may not be in the DEGs. When these CTGs were mapped to the aforementioned DEG gene set (Supplementary Tables S7–S9), leading to acquisition of 107, 470, and 222 differentially expressed CTGs. Matching these differentially expressed CTGs to DE-miRNAs again resulted in just 158, 855, and 297 DE-miRNA-CTG pairs in PS *vs* TS, PS *vs* SS, and TS *vs* SS comparisons, respectively (Supplementary Tables S16–S18). Among these pairs, 33, 109, and 107 pairs had a significantly positive correlation and 78, 307, and 119 pairs had no significant correlation, while 47, 439, and 71 miRNA/CTG pairs had a significantly negative correlation with Pearson correlation coefficients varying from −0.812 to −0.991 in PS *vs* TS, PS *vs* SS, and TS *vs* SS, respectively (Supplementary Tables S16–S18 and Figure 3C). After removing duplicates, the miRNA-CTG pairs comprised 23, 128, and 25 DE-miRNAs and 35, 230, and 65 corresponding CTG, respectively (Supplementary Tables S19–S21). The significant inverse relationships between DE-miRNAs and their CTGs in profiles indicate these miRNAs exert the strongest regulation on these CTGs. Other DE-miRNAs and CTGs, which were in relatively large quantity and did not exhibit significant pairwise inverse relationships, might be subjected to more complicated multiple regulation by multiple miRNAs, and most likely by *TFs*, constituting extremely complicate regulatory relationships and networks in the genome. Notably, complicated interaction could result in positive correlated pairs. For example, Potri.002G033800 was a target of miR6425-x, and they showed a negative correlation in our data. However, Potri.002G033800 is also a target gene of novel-m0409-5p and novel-m0409-5p, but they had a positive correlation in our data. Presumably, one prevalent miRNA can change the correlation between its target and other miRNAs to positive. There are certainly other scenarios that can result in positive correlation of miRNAs and their targets.

### Regulatory relationships of DE-miRNA and CTGs

**Figure 4:**
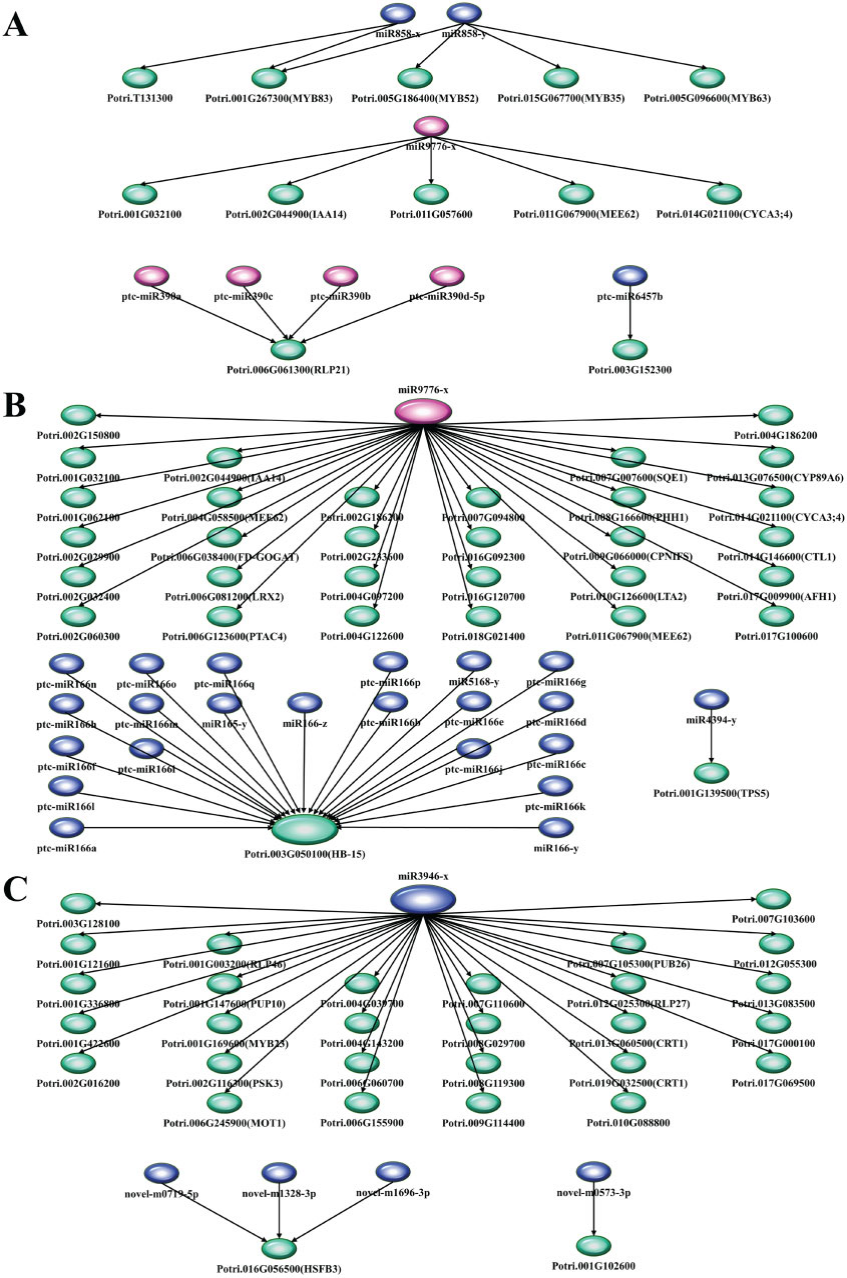
The typical regulatory relationships of DE-miRNAs and CTGs. (A) The typical regulatory relationships of DE-miRNAs and CTGs in PS *vs* TS. (B) The typical regulatory relationships of DE-miRNAs and CTGs in PS *vs* SS. (C) The typical regulatory relationships of DE-miRNAs and CTGs in TS vs SS. Pink represents up-regulated miRNA, Blue represents down-regulated miRNA, Green represents CTGs.

Among the above significantly and negatively related DE-miRNA-CTG pairs, 6, 27, and 6 miRNA families had more than one CTGs, while one, four, and one miRNA family have only one CTG in PS *vs* TS, PS *vs* SS, and TS *vs* SS, respectively (Supplementary Tables S18–S20). For example, in PS *vs* TS, miR858 and miR9776-x had five CTGs, Potri.T13100, *MYB83* (Potri.001G267300), *MYB52* (Potri.005G186400), *MYB63* (Potri.005G096600), and *MYB35* (Potri.015G067700), about 14.3% of all CTGs (Figure 4A); in PS *vs* SS, miR9776-x had 30 CTGs, including *CYCA3; 4* (Potri.014G021100), Potri.002G032400, and *IAA14* (Potri.002G044900), accounting for 13.0% of all CTGs (Figure 4B); miR3946-x had 28 CTGs, including *CRT1* (Potri.013G060500 and Potri.019G032500), *RLP46* (Potri.001G003200), and *MYB23* (Potri.001G169600), accounting for 43.0% of all CTGs in TS *vs* SS (Figure 4C). In addition, 8, 26, and 11 DE-miRNAs were associated with only one CTG, such as ptc-miR6457b and CTG Potri.003G152300 in PS *vs* TS, miR4394-y and CTG *TPS5* (Potri.001G139500) in PS *vs* SS, and novel-m0573-3p and CTG Potri.001G102600 in TS *vs* SS, respectively (Figure 4). The lower connectivity of these miRNAs in their regulatory networks suggests that they are not powerful effectors.

Furthermore, multiple DE-miRNAs had one CTG. For example, *RLP21* (Potri.006G061300) was repressed by four members of the ptc-miR-390 family in PS *vs* TS (Figure 4A). *HB-15* (Potri.001G188800) was repressed by 19 members of the ptc-miR166 family, ptc-miR5168-y, and ptc-miR165-y in PS *vs* SS (Figure 4B). In addition, in TS *vs* SS (Figure 4C), *HSFB3* (Potri.016G056500) was repressed by novel-m1696-3p, novel-m1328-3p, and novel-m0719-5p. The above results suggest that these DE-miRNAs performed various biological functions through repressing one CTG, multiple CTGs, or multiple DE-miRNAs involved in the same or different biological processes in diverse phases of wood formation.

### Functions of DE-miRNAs in diverse phases of wood formation

**Figure 5:**
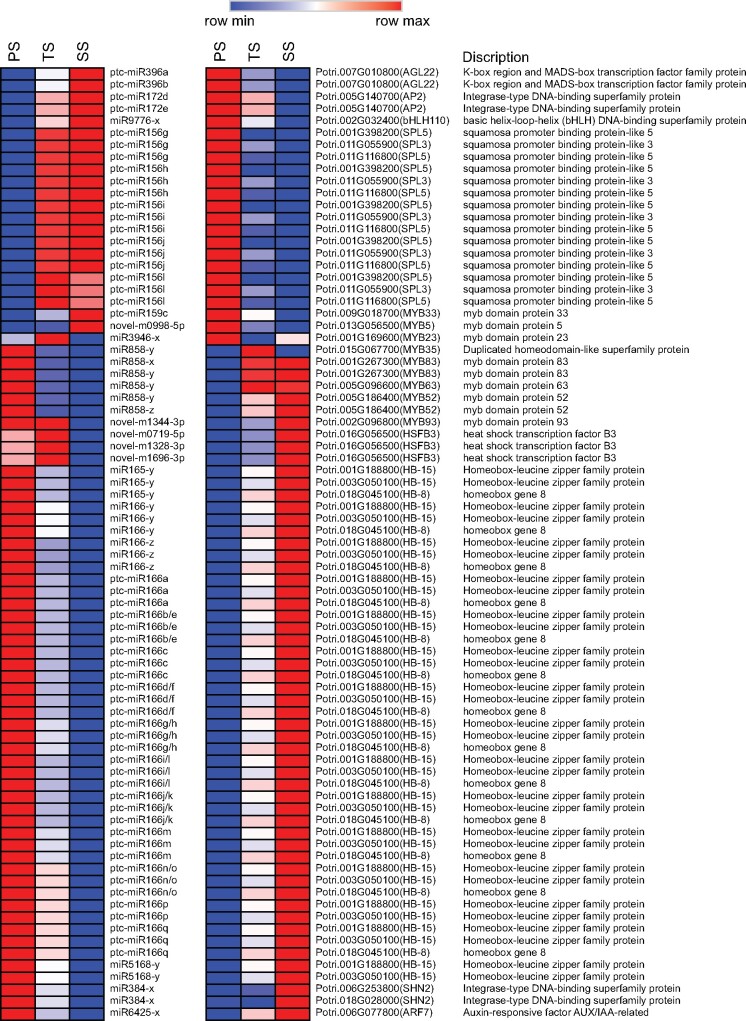
Heatmap of DE-miRNAs and *TFs* in CTGs with inverse relationships in their expression profiles from primary stem (PS) to transition stem (TS) and then to secondary stem (SS).

Generally, the biological functions of miRNAs are manifested through negatively regulating their CTGs by either mRNA degradation or translational suppression based on sequence complementarity with their target(s) (Brodersen *et al.* 2008). Thus, we deduced the biological functions of these above DE-miRNAs by analyzing the functions of their significantly and negatively regulated CTGs. According to the functional annotation of these CTSs, we found 5, 30, and 4 CTGs encoding TFs, which are CTGs of 3, 37, and 6 corresponding DE-miRNAs and formed 6, 142, and 6 DE-miRNA-CTG pairs, in PS *vs* TS, PS *vs* SS, and TS *vs* SS, respectively (Supplementary Tables S19–S21). Some of these *TFs* have been reported to play important roles in diverse phases of wood formation. For example, *IAA14* (Potri.002G044900), which is the miR9776-x CTG and significantly up-regulated in PS compared with that in TS and SS (Figure 5), is involved in auxin signal transduction and vascular development by interacting with *ARF5* (Liu *et al.* 2015b). Moreover, multiple *TFs* in the CTGs of DE-miRNAs in PS *vs* SS that were significantly up-regulated in PS compared with those in SS were primarily involved in cell division and expansion, processes in the beginning phase of wood formation. For example, the *SPL2* (Potri.018G149900), *SPL10* (Potri.014G057800), *SPL11* (Potri.002G142400), *SPL3* (Potri.011G055900), and *SPL5* (Potri.011G116800) CTGs of the miRNA156 family have been reported to regulate lateral root growth by directly regulating *AGL79* and shoot development in the vegetative phase (Shikata *et al.* 2009; Usami *et al.* 2009; Preston and Hileman 2013; Gao *et al.* 2017). ptc-miR172d and ptc-miR172e CTG *AP2* (Potri.005G140700) are involved in cell division and elongation (Huijser and Schmid 2011). ptc-miR159c CTG *MYB33* (Potri.009G018700) promotes cell division in the root meristem by accelerating the cell cycle (Xue *et al.* 2017). In addition, *AGL22* (Potri.007G010800), the CTG of ptc-miR396a and ptc-miR396b, is involved in the transition from the vegetative state to flowering (Bechtold *et al.* 2016). The miR9776-x CTG *bHLH110* (Potri.002G032400), together with *AtIBH1* and *PRE1*, constitute a tri-antagonistic *bHLH* system and competitively regulates cell elongation under brassinosteroids (BRs), gibberellins (GAs), and developmental phase-dependent signals (Ikeda *et al.* 2012). In TS, we also found some *TFs* that were up-regulated compared with those in PS or SS and involved in the cell wall modification and SCW biosynthesis processes of the middle phases of wood formation. For example, *MYB83* (Potri.001G267300), the CTG of miR858-x and miR858-y, has been reported to directly regulate secondary wall biosynthesis (Zhong *et al.* 2008). *MYB63* (Potri.005G096600), *MYB52* (Potri.005G186400), and *MYB35* (Potri.015G067700), CTGs of miR858-y, activate lignin biosynthesis (Zhou *et al.* 2009), participate in SCW biosynthesis in xylary fibers (Zhong *et al.* 2008), and regulate callose deposition around pollen mother cells (Tsugama *et al.* 2017), respectively (Figure 5). *MYB5* (Potri.013G056500), a CTG of novel-m0998-5p, can form MYB–bHLH–WDR (MBW) TF complexes together with TT2 and TTG1 to regulate cuticle biosynthetic pathways (Li *et al.* 2020a) and activate flavonoid pathway genes, such as *PAL* and *CHS* (Liu *et al.* 2014), of which *PAL* encodes the first enzyme of lignin biosynthesis pathway (Dixon and Barros 2019). In addition, we observed that some *TFs* of DE-miRNA CTGs were up-regulated in SS compared with those in PS or TS and participated in some typical processes of the late phase of wood formation. For example, *SHN2* (Potri.006G253800 and Potri.018G028000), which was the CTG of miR384 that was significantly up-regulated in SS compared with that in PS (Figure 5), is a top-level master switch in the hierarchical transcriptional regulation network of SCW formation, up-regulating the expression of genes related to cellulose and hemicellulose biosynthesis and repressing the expression of genes involved in lignin biosynthesis and PCD in poplar (Liu *et al.* 2017). *MYB83* (Potri.001G267300), which was the CTG of miR858-x and miR858-y and up-regulated in SS compared with that in PS, is a master switch that regulates multiple downstream *TFs* and structural genes related to SCW biosynthesis and PCD (Zhong *et al.* 2008; Ko *et al.* 2014). miR858-y CTG *MYB63* (Potri.005G096600), which was up-regulated in SS compared with that in PS, is a direct target of *MYB46* and functions as a direct transcriptional activator of lignin biosynthesis (Zhong *et al.* 2008; Ko *et al.* 2014). miR858-z CTG *MYB52* (Potri.005G186400), which was significantly up-regulated in SS compared with that in PS, is a downstream *TF* of *MYB46* that activates genes involved in SCW biosynthesis (Ko *et al.* 2009). Moreover, we found that some *TFs*, up-regulated in SS compared with those in TS, are related to various processes of the late phases of wood formation. For example, miR6425-x CTG *ARF7* (Potri.006G077800) indirectly regulates cell wall synthesis and remodeling by activating *MUS* and *MUL*, two kinase-inactive RLKs, under auxin (Xun *et al.* 2020). *MYB23* (Potri.001G169600), a CTG of miR3946-x, is a homologous gene of *MYB52* and plays roles in regulating cuticle biosynthetic pathways (Li *et al.* 2020a). *HB-15* (Potri.001G188800 and Potri.003G050100), a CTG of the miR165-y, miR5168-y and miR166 family, up-regulates genes involved in cellulose biosynthesis and down-regulates lignification- and metabolite-related genes (putative laccase, cinnamoyl CoA reductase, chalcone synthase, putative pectin methyltransferases, and pectin esterase) (Kim *et al.* 2005; Du *et al.* 2011). *HB-8* (Potri.018G045100), a CTG of the miR165-y and miR166 families, promotes vascular cell differentiation and increases the production of lignified tissues (Baima *et al.* 2001). In addition, we found that *MYB93* (Potri.002G096800), a CTG of novel-m1344-3p, and *HSFs* (Potri.016G056500), CTGs of novel-m0719-5p, novel-m1328-3p, and novel-m1696-3p, were up-regulated in SS compared with those in TS, while the functions of these *TFs* in wood formation remain unknown.

**Figure 6:**
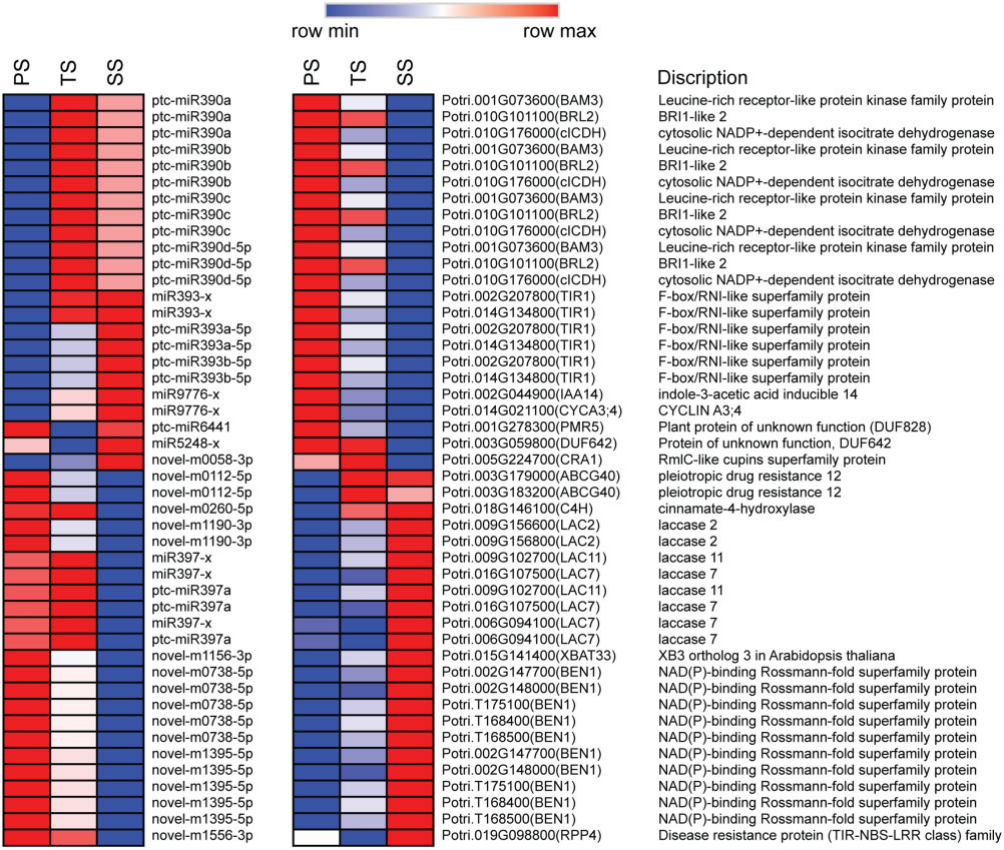
Expression profiles of miRNAs and CTGs which were structural mRNA-encoding genes related to wood formation of primary stem (PS) to transition stem (TS) and then to secondary stem (SS).

Moreover, we found that some CTGs of DE-miRNAs were structural genes related to diverse processes of wood formation. For example, the miR9776-x CTG *CYCA3; 4* (Potri.014G021100), miR393 family CTG *T1R1* (Potri.014G134800 and Potri.002G207800), and ptc-miR390a CTGs, *BAM3* (Potri.001G073600), *ICDH* (Potri.010G176000), and *BRL2* (Potri.010G101100), which have been reported to participate in cell division, expansion, and differentiation, respectively (Masubelele *et al.* 2005; Deyoung *et al.* 2006; Doğramacı *et al.* 2015; Belén *et al.* 2018), were significantly up-regulated in PS compared with those in SS or TS (Figure 6). ptc-miR6441 CTG *PMR5* (Potri.001G278300), which affects pectin composition (Vogel *et al.* 2004); novel-m0058-3p CTG *CRA1* (Potri.005G224700), which regulates lignification and flavonoid production (Laffont *et al.* 2010), and miR5248-x CTG *DUF642* (Potri.003G059800), which is a pectin methyl esterase and contributes to the modification of cell wall properties (Cruz-Valderrama *et al.* 2019), were up-regulated in TS compared with those in PS or SS (Figure 6). Furthermore, novel-m0260-5p CTG *C4H* (Potri.018G146100), novel-m1190-3p CTG *LAC2* (Potri.009G156600 and Potri.009G156800), and miR397-x CTGs *LAC7* (Potri.016G107500 and Potri.006G094100) and *LAC11* (Potri.009G102700) related to lignin biosynthesis and deposition (O'Malley *et al.* 1993; Zhao *et al.* 2013; Sykes *et al.* 2015), miR6425-x CTG *ADF6* (Potri.002G038800) involved in the processes of cellulose elongation and SCW formation during fiber development (Wang *et al.* 2009), novel-m0738-5p and novel-m1395-5p CTG *BEN1* (Potri.002G147700, Potri.002G148000, Potri.T168400, Potri.T168500, and Potri.T175100), novel-m1156-3p CTG *XBAT33* (Potri.015G141400), novel-m0112-5p CTG *ABCG40* (Potri.003G183200 and Potri.003G179000), and novel-m1556-3p CTG *RPP4* (Potri.019G098800) involved in PCD (Aoyagi *et al.* 1998; Knoth *et al.* 2007; Huang *et al.* 2013; Bouwmeester *et al.* 2015), were up-regulated in SS compared with those in PS or TS (Figure 6).

We also found that the functions of some DE-miRNAs have not been clarified to date in wood formation, although their expression levels exhibited extreme alternations among PS, TS, and SS (Supplementary Tables S19–S21), suggesting these DE-miRNAs might play roles in specific phases of wood formation. For example, miR858-x and novel-m1201-5p were significantly down-regulated in PS compared with those in TS and SS, with their corresponding CTGs Potri.T131300 and Potri.010G088200 showing contradictory changes. The expression levels of miR9776-x and ptc-miR172d and their corresponding CTGs Potri.001G062100 and Potri.009G009100 showed significant reverse alternations in TS compared with those in PS and SS. However, the functions of these miRNAs in different phases of wood formation require further study.

**Figure 7:**
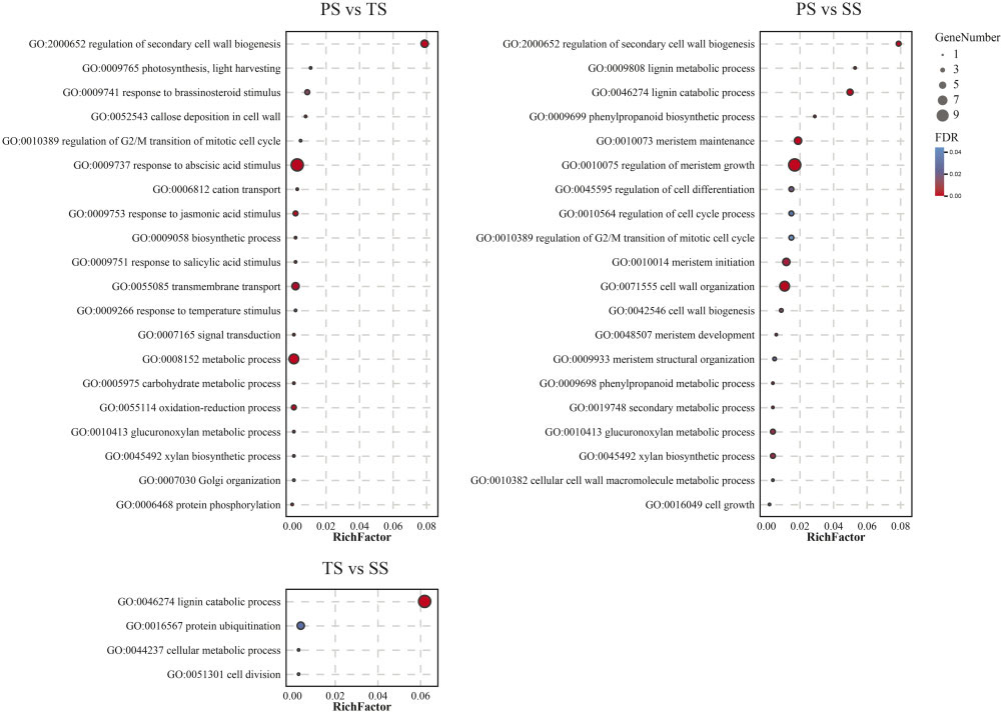
Typical biological processes of significant GO terms from CTGs of DE-miRNAs. The *x*-axis represents Rich Factor. The *y*-axis represents GO term. FDR ≤ 0.05 indicates significant enrichment. The size of each circle represents number of gene, and color represents FDR value.

To further determine the biological roles of the above DE-miRNAs in wood formation, we also performed GO and KEGG analyses of their corresponding CTGs in PS *vs* TS, PS *vs* SS, and TS *vs* SS. As shown in Figure 7 and Supplementary Tables S22–S24, some significantly enriched biological processes in PS *vs* TS were the typical processes of the beginning and middle phases of wood formation. For example, “regulation of G2/M transition of mitotic cell cycle” (GO:0010389) was involved in the cell division of the meristem, while the “regulation of SCW biogenesis” (GO: 2000652), “callose deposition in cell wall” (GO:0052543), “glucuronoxylan metabolic process” (GO:0010413), and “xylan biosynthetic process” (GO:0045492) were related to the SCW initially formation (Figure 7 and Supplementary Table S22). In addition, the most two significantly enriched cellular component were “membrane” (GO:0016020) and “nucleus” (GO:0005634). And “sequence-specific DNA binding transcription factor activity” (GO:0003700) and “DNA binding” (GO:0003677) were the most two significantly enriched molecular functions. In PS *vs* SS, some significantly enriched processes, such as “regulation of meristem growth” (GO:0010075), “regulation of cell differentiation” (GO:0045595), and “primary shoot apical meristem specification” (GO:0010072), and so on (Figure 7 and Supplementary Table S23), were the typical processes of the beginning phase of wood formation, while the “regulation of SCW biogenesis” (GO:2000652), “cell wall organization” (GO:0071555), and “cellular cell wall macromolecule metabolic process” (GO:0010382), and so on (Figure 7 and Supplementary Table S23), were the typical processes of secondary phases of wood formation. The most two significantly enriched cellular components were “nucleus” (GO:0005634), and “membrane” (GO:0016020). And “DNA binding” (GO:0003677) was the most significantly enriched molecular function. Among these significantly enriched processes in TS *vs* SS (Figure 7 and Supplementary Table S24), “cell division” (GO:0051301) was related to the primary and middle phase of wood formation. And “lignin catabolic process” (GO:0046274) was involved in the late phase of wood formation. The significantly enriched cellular component was “extracellular region” (GO:0005576). And “copper ion binding” (GO:0005507) and “protein binding” (GO:0005515) were the most two significantly enriched molecular functions.

The results of KEGG analysis showed multiple significantly enriched pathways related to typical pathways of the different phases of wood formation in the PS *vs* TS, PS *vs* SS, and TS *vs* SS (Supplementary Table S25). In PS *vs* TS, “Plant hormone signal transduction (ko04075)” and “Photosynthesis-antenna proteins (ko00196)” are related to cell division and differentiation activity (Niinemets and Lukjanova 2003; Kawamura and Takeda 2004; Evers *et al.* 2006; Heisler *et al.* 2010), which were the typical pathways of cells in the beginning phase of wood formation. We also found that some significantly enriched pathways in PS *vs* SS were typical pathways of the late phase of wood formation (Supplementary Table S25), such as “Phenylalanine metabolism (ko00360)” related to cell wall lignification (Rinaldi *et al.* 2016), “Biosynthesis of secondary metabolites (ko01110)” implicated in cellulose and lignin biosynthesis (Roeschlin *et al.* 2017; Xie *et al.* 2018), and “Flavonoid biosynthesis (ko00941)” involved in cell wall stabilization (Vidot *et al.* 2018), while “Photosynthesis-antenna proteins (ko00196)” was the typical pathway of the beginning phase of wood formation (Supplementary Table S25). In addition, we obtained some significantly enriched pathways belonging to the middle and late phases of wood formation in TS *vs* SS. For example, “Glycine, serine and threonine metabolism (ko00260),” which is reported to be related to cell wall synthesis and cell wall remodeling pathways (Preeti *et al.* 2018), was the typical pathway in the middle phase of wood formation, while the “Biosynthesis of secondary metabolites (ko01110)” and “Phenylalanine metabolism (ko00360)” are typical pathways in the late phase of wood formation (Rinaldi *et al.* 2016; Roeschlin *et al.* 2017; Xie *et al.* 2018).

Together, these results demonstrate that the DE-miRNAs of PS *vs* TS, PS *vs* SS, and TS *vs* SS played various roles in the diverse phases of wood formation by regulating CTGs, which are regulators and structural genes involved in diverse biological processes and pathways of wood formation.

### Validation of miRNAs and their CTG expressions using RT-PCR

**Figure 8:**
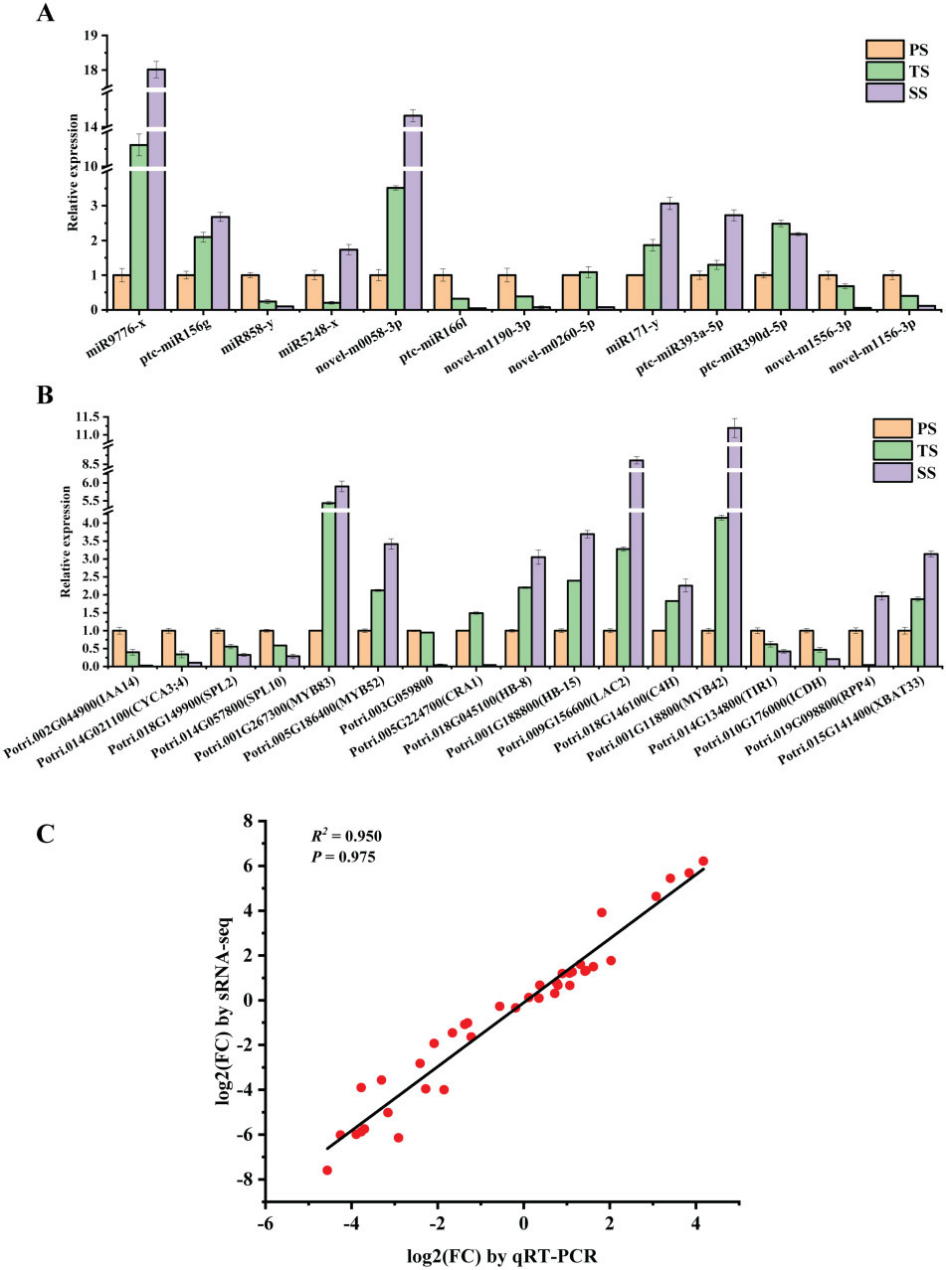
The expression levels of some representative miRNAs and CTGs. (A) the relative expression level distribution of 13 selected miRNAs (8 known miRNA and 5 novel miRNA) determined by RT-PCR in primary stem (PS), transition stem (TS), and secondary stem (SS). (B) the relative expression level distribution for the 17 genes targeted by 11 miRNAs. The expression levels of miRNAs and their mRNA targets were normalized to the level of 5.8S rRNA and *β-actin*, respectively. Each value is represented by mean ± SD of the triplicates of three biological replicates. (C) Linear regression analysis for comparing the results of RT-qPCR and sRNA-seq. The *y*-axis represents the fold-change values by sRNA-seq, and the *x*-axis represents the fold-change values by qRT-PCR. The *R*^2^ is 0.950 and correlation coefficient equals to 0.975. Solid lines represent linear regression lines.

To validate the expression levels of miRNAs obtained using sRNA-seq and their expression dynamics among PS, TS, and SS, the expression levels of eight known miRNAs (miR9776-x, ptc-miR156g, miR858-y, miR5248-x, ptc-miR166l, miR171-y, ptc-miR393a-5p, and ptc-miR390d-5p) and 5 novel miRNAs (novel-m0058-3p, novel-m1190-3p, novel-m0260-5p, novel-m1556-3p, and novel-mm1156-3p) were analyzed by qRT-PCR using the same RNA sources as those for sRNA-seq analysis. The relative transcript levels of these examined miRNAs by qRT-PCR showed good agreement with those obtained by sRNA-seq (Figure 8A). Moreover, there was a significant regression line with *R*^2^ equal to 0.95 between miRNA expression values obtained by qRT-PCR and sRNA-seq (Figure 8C), indicating that the expression values of miRNA were obtained from two analysis methods were in good agreement. These results confirmed the robustness and reliability of the sRNA-seq results.

To confirm the negative correlations between the miRNAs and their CTGs obtained by integrated analysis of the sRNA and mRNA profiles, the expression levels of the CTGs of miRNAs in PS, TS, and SS were also investigated by qRT-PCR. The expression levels of *IAA14* (Potri.002G044900), *CYCA3; 4* (Potri.014G021100), *SPL2* (Potri.018G149900), *SPL10* (Potri.014G057800), *TIR1* (Potri.014G134800), *MYB83* (Potri.001G267300), *MYB52* (Potri.005G186400), *HB-8* (Potri.018G045100), *HB-15* (Potri.001G188800), *LAC2* (Potri.009G156600), *XBAT33* (Potri.015G141400), *ICDH* (Potri.010G176000), *CRA1* (Potri.005G224700), *DUF642* (Potri.003G059800), *RPP4* (Potri.019G098800), and *C4H* (Potri.018G146100) exhibited opposite trends to the corresponding miR9776-x, ptc-miR156g, ptc-miR393-5p, miR858-y, ptc-miR166l, novel-m1190-3p, novel-m1156-3p, ptc-miR390d-5p, novel-m0058-3p, miR5248-x, novel-m1556-3p, and novel-m0260-5p in PS, TS, and SS, respectively (Figure 8, A and B). However, we also found that the expression levels of miR171-y and CTG *MYB42* (Potri.001G118800) exhibited the same change trend from PS to SS (Figure 8, A and B), suggesting a positive correlation between this miRNA-CTG pair and demonstrating the presence of other regulators such as *MYB46* (Geng *et al.* 2020), together with miRNA, potentially involved in controlling the expression of *MYB42*. Together, these results also demonstrated that the identified miRNAs involved in the diverse phase of wood formation were generally negatively related to their CTGs.

## Discussion

Wood formation comprises several continuous biological processes (Zhong *et al.* 2010) that require multiple molecular regulatory mechanisms to synergistically regulate the expression of many genes related to various processes of wood formation (Zhong *et al.* 2008; Puzey *et al.* 2012; Xu *et al.* 2014). Although numerous miRNAs involved in the regulation of specific biological processes in wood formation have been identified thus far (Du *et al.* 2011; Wang *et al.* 2011b; Lu *et al.* 2013; Zhao *et al.* 2015; Chen *et al.* 2016, 2018; Xiao *et al.* 2017), how many miRNAs exist and the molecular functions of these miRNAs in the diverse processes of wood formation remain unclear. The vertical segments of the less than 1-year-old poplar that include all processes of wood formation and that can be divided into three phases—beginning, middle, and late phases of wood formation (Prassinos *et al.* 2005; Dharmawardhana *et al.* 2010)—have been used to investigate the dynamics of the molecular regulatory mechanisms underlying diverse phases of wood formation (Dharmawardhana *et al.* 2010). In this study, to uncover the dynamics of miRNAs and their biological functions underlying diverse phases of wood formation, we performed integrated analysis of the mRNA and sRNA expression profiling of PS, TS, and SS in less than 1-year-old poplar trunks by Solexa sequencing.

### miRNA length and expression variation among diverse phases of wood formation

Through sRNA high-throughput sequencing technology, we generated 15,687,101, 15,980,089, and 1,226,233 reads from PS, TS, and SS libraries and finally identified 12,237,495, 11,639,629, and 9,336,272 sRNAs, respectively (Table 1). These results demonstrate that the *P. trichocarpa* woods during diverse phases contain large and diverse sRNA populations, a finding similar to previous study findings in *P. balsamifera* (Barakat *et al.* 2007) and Chinese fir (Wan *et al.* 2012). Moreover, we observed that 21-nt sRNAs were the most numerous, comprising approximately 50.75% of the total RNAs, with 24-nt sRNAs being the second most frequent, averaging approximately 14.00%. These distribution patterns of sRNA lengths in diverse phases of wood formation in *P. trichocarpa* were also found in grapevine (Wang *et al.* 2011a), cotton (Xie *et al.* 2015), Chinese white poplar (Ren *et al.* 2013), and Norway spruce (*Picea abies*) (Yakovlev *et al.* 2010). However, it was notable that the abundances of 21- and 24-nt sRNAs exhibited significant differences among PS, TS, and SS. The differences in the 21- and 24-nt sRNA abundances were also observed in *P. tomentosa* under phosphate starvation (Wei *et al.* 2019), different development states of cambium in *Cunninghamia lanceolate* (Wan *et al.* 2012), and between angiosperms and gymnosperms (Wan *et al.* 2012). These results demonstrate that the lengths of sRNAs and abundances of major sRNAs vary among plant species, developmental states, and tissues of the same plant.

Because most plant sRNAs are produced as 21- to 24-nt RNA molecules due to the activities of Dicers (*DCLs*), Argonautes (*AGOs*), RNA-dependent RNA polymerases (*RDRs*), and SUPPRESSOR OF GENE SILENCING2/3 (*SGS2/3*) (Peragine *et al.* 2004; Dolgosheina *et al.* 2008), we suspected differences in the expression of the above genes among PS, TS, and SS. We observed that the expression of *DCL4* (Potri.006G188800), *DCL2* (Potri.008G075900 and Potri.010G181400), *RDR2* (Potri.015G073700), *AGO4* (Potri.006G025900), *AGO6* (Potri.014G159400), *AGO5* (Potri.009G001500 and Potri.001G213700), *AGO10*(Potri.010G081300 and Potri.008G158800), *AGO7* (Potri.010G163800), *AGO2* (Potri.012G118700 and Potri.015G117400), and *SGS3* (Potri.003G187900, Potri.003G188100, and Potri.003G188200) obviously varied among PS, TS, and SS (Supplementary Tables S7–S9). However, no consistencies were found between the expression abundances of the above genes and those of 21- to 24-nt RNA molecules among PS, TS, and SS. We speculated that these genes might have post-transcription or post-translation modifications. Interestingly, *AGO2* (Potri.015G117400) was found to be regulated by miR1167-y in PS *vs* SS. In summary, these results further demonstrate significant differences in the molecular mechanism of the sRNA biogenesis pathways among diverse phases of wood formation.

### miRNAs identified from diverse phases of wood formation

We identified 892 (361 existing miRNAs and 531 conserved miRNAs), 872 (345 existing miRNAs and 527 conserved miRNAs), and 882 (334 existing miRNAs and 548 conserved miRNAs) known miRNAs from 491, 478, and 503 miRNA families, and 1727, 1723, and 1597 novel miRNAs in PS, TS, and SS, respectively (Figure 2C). Among these miRNAs, nine conserved miRNAs, such as miR9776, miR7741, miR6265, and miR4394 (Supplementary Table S26), had not been previously reported in *Populus* or other plant species, and 760 miRNAs, such as miR8664-y, miR7729-y, miR6277-y, and miR5815-x, belonged to low-expression miRNAs (TPM < 1) (Supplementary Table S2). These results also demonstrate that the sRNA high-throughput sequencing approach is suited for identifying more species-specific miRNAs or miRNAs with very low expression in plants. In addition, we found that only 56 (2.14%), 58 (2.24%), and 55 (2.22%) miRNAs showed TPM > 1000 on average, accounting for 99.65, 99.26, and 98.56% of the total TPM of miRNAs in PS, TS, and SS, respectively (Supplementary Tables S2 and S3). These results were consistent with those of previous reports that the plant sRNAs primarily comprise many miRNAs with low-level expression and few miRNAs with high-level expression (Brodersen *et al.* 2008).miRNA families that had high-level expression in one or two stages might play important roles in this or these stages. For example, ptc-miR166 family had the highest abundance in PS and TS with 21,349,509 and 8,730,386 TPM, respectively. They could inhibit expression of many of their target genes in PS and TS, and we found that their target gene family (*HD-ZIP*) had a low-level expression in PS and TS; The *HD-ZIP* family members function in cell differentiation during secondary growth (Du *et al.* 2011), and they were inhibited in PS and TS. Similarly, ptc-miR396 family, with 5,034,301 TPM, was the highest expressed miRNA family in SS. Its target gene, *AGL22*(*SVP*), which is known to function in controlling meristem development during vegetative growth and flower development (Gregis *et al.* 2013), was inhibited and exhibited a low level of expression in SS.

Plant miRNAs only have a few mRNA targets (Addo-Quaye *et al.* 2008). However, our data demonstrated 1867 (921, 2639, and 2042) CTGs, targets of 190 (114, 306, and 152) DE-miRNAs on average in poplar stems (PS, TS, and SS), approximately 4% of the protein-coding genes of poplar and greater than approximately 1 and 3% of the protein-coding genes in subcultured *Taxus* cells and *Arabidopsis*, respectively (Addo-Quaye *et al.* 2008; Zhang *et al.* 2015). These results indicate that our miRNA data had excellent coverage.

We predicted 158, 855, and 297 DE-miRNA-CTG pairs between the 114, 306, and 152 DE-miRNAs and 921, 2639, and 2042 corresponding CTGs in PS *vs* TS, PS *vs* SS, and TS *vs* SS, respectively (Figure 3, A and B and Supplementary Tables S4–S9). Among these DE-miRNA-CTG pairs, 47, 439, and 71 pairs showed a significantly negative correlation, 33, 109, and 107 pairs showed a significantly positive correlation, and 78, 307, and 119 pairs showed no significant correlation, representing 42.5, 22.4, and 35.5% of the total DE-miRNA-CTG pairs on average, respectively (Supplementary Tables S16–S18). These results demonstrate that the negatively related DE-miRNA-CTG pairs numbered about twice as high as the positively related pairs, a finding that is not consistent with the findings in *Taxus* and the tomato (Lopez-Gomollon *et al.* 2012; Zhang *et al.* 2015), suggesting the differently related miRNA-mRNA pairs vary among plant species or different tissues of the same plant. In addition, because a gene can be regulated by multiple regulatory factors, the inhibitory effects of a miRNA on its target gene can be attenuated or masked by other regulatory factors; thus, the relationship between a miRNA and target gene does not necessarily show a negative correlation. For example, *LAC4* is not only repressed by ptr-miR397a but also activated by *MYB58* and *MYB63* in poplar (Lu *et al.* 2013). In this study, perhaps due to the *MYB46* and *MYB83* activating effects on *MYB42* (Zhong *et al.* 2008; Geng *et al.* 2020), miR171-y and its target gene *MYB42* (Potri.001G118800) exhibit a positive correlation.

Although these DE-miRNAs with nonnegative relation of their CTGs could function in the biological processes of plants according to a previous report (Nikovics *et al.* 2006), the functions of these DE-miRNAs could not be determined because their target genes were not accurately identified by integrated analysis of the miRNA and mRNA expression profiles due to the interference effects of other regulatory factors. Thus, in this study, we did not further analyze these positively or not significantly related DE-miRNA-CTG pairs and only focused on the significantly negatively related DE-miRNA-CTG pairs. Notably, these negatively related DE-miRNA-CTG pairs comprised 23, 128, and 25 DE-miRNAs, and 35, 230, and 65 corresponding CTGs (Supplementary Tables S19–S21), representing only 20.17, 41.83, and 16.45% of the total DE-miRNAs and 0.70, 2.19, and 1.36% of the total DEGs in PS *vs* TS, PS *vs* SS, and TS *vs* SS, respectively. These results suggest that miRNA regulation probably accounts for a small part of the molecular regulatory mechanisms underlying diverse phases of wood formation.

### Functions of previously identified miRNAs in diverse phases of wood formation

Previous studies in tree species have revealed that miRNAs are implicated in multiple biological processes of plant growth and development by regulating their target genes (Donaire *et al.* 2011). In this study, we also revealed that multiple miRNAs, which have been reported previously to play important roles in multiple processes of plant growth and development, are involved in different phases of wood formation.

**Figure 9:**
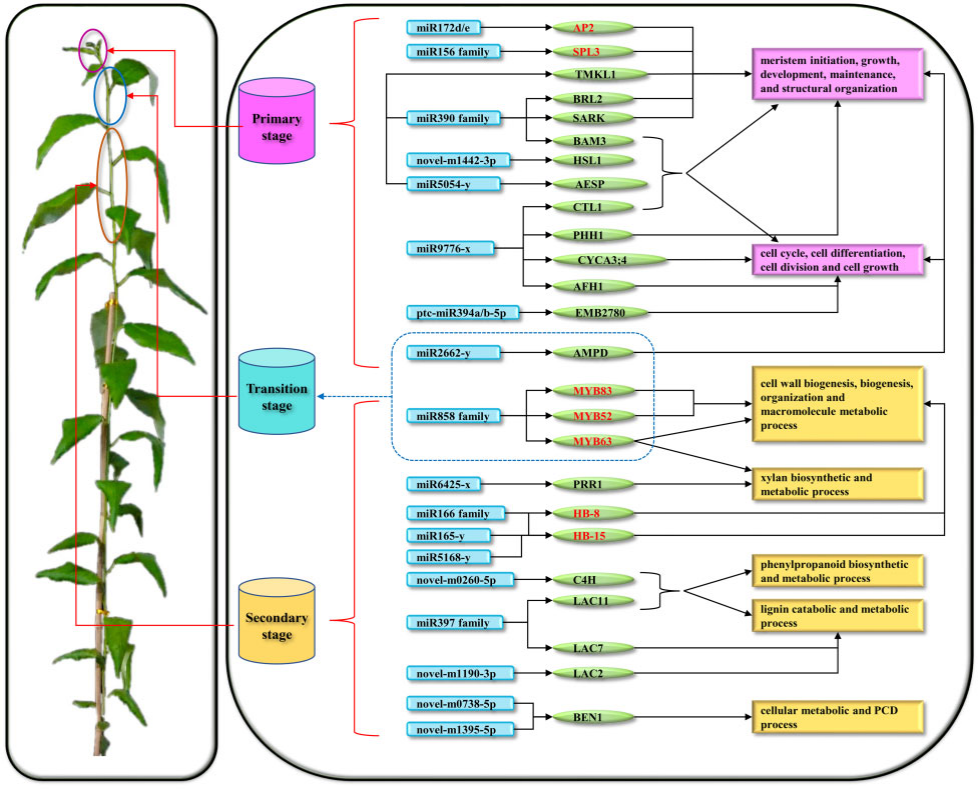
The identified miRNAs and target genes that were associated wood formation. Each blue arc edge rectangle represents a miRNA; each green ellipse contains a target gene (red font means TF identified in this study); Each violet or yellow rectangle shows the function involved in primary or secondary stage of wood formation. The dotted line rectangle represents miRNAs and targets participate in transition stage of wood formation.

For example, the miRNA156 family was significantly down-regulated in PS compared with that in SS, suggesting that the miRNA156 family participated in the beginning phase of wood formation by alleviating their inhibitory effects on target *SP3* (Potri.011G055900) related to the meristem development process (Figure 9). miR156 regulates cell division and elongation by down-regulating the *SPL* family in vascular cambium transition from dormancy to the active growth phase in *C. lanceolate* (Qiu *et al.* 2015) and the juvenile-to-adult transition in *Arabidopsis* and maize (Wu *et al.* 2009; Zhang *et al.* 2009b). In addition, miRNA156 is involved in lateral root growth and is responsive to auxin signaling by repressing *SPLs* in *Arabidopsis* (Yu *et al.* 2015). Ptc-miR172d and ptc-miR172e were observed to be involved in the beginning phase of wood formation by significantly reducing the inhibition effects on target *TFs AP2* (Potri.005G140700) (Figure 9), which related to the meristem maintenance process, in PS compared with those in SS. The functions of ptc-miR172d and ptc-miR172e in this study were somewhat similar to that of cln-miR172, which regulates cell division and elongation by alleviating the inhibitory effect on its target *TF AP2* in the transition from the dormancy of vascular cambium in *C. lanceolate* (Qiu *et al.* 2015), but was different from that of miR172 in *Arabidopsis*, being involved in the autonomous flowering pathway by regulating its target *TF TOE1* (Jung *et al.* 2007). These results suggest that miR172 may be involved in multiple biological processes in plant growth and development by regulating the same or different target *TFs*.

miR858 family can regulate *MYB81*, *MYB97*, *MYB120*, *MYB65*, and *MYB104* orthologs in *Salvia miltiorrhiza* (Li and Lu 2014), a negative regulator of anthocyanin biosynthesis, by repressing the target *AaMYBC1* in red-colored kiwifruit (Li *et al.* 2020b), mediating the cleavage of *SlMYB7-like* and *SlMYB48*-like *TFs* in *Solanum lycopersicum* (Jia *et al.* 2015), regulating *VvMYB114* to promote anthocyanin and flavanol accumulation in grapes (Tirumalai *et al.* 2019), regulating the homologous *MYB2* gene in *Arabidopsis* trichome and cotton fiber development (Guan *et al.* 2014), and interfering with the *Heterodera schachtii* parasitism of *Arabidopsis* by repressing *MYB83* (Piya *et al.* 2017). In this study, we found that miRNA858 family participated in the SCW biosynthesis, xylan biosynthetic and metabolic process of the middle and late phases of wood formation by significantly alleviating the inhibitory effects on the target *TFs MYB52* (Potri.005G186400), *MYB83* (Potri.001G267300), and *MYB6*3 (Potri.005G096600) in TS and SS compared with those in PS. To our best knowledge, this report is the first to identify miRNA858s as being involved in wood formation by regulating the key *TFs* (*MYB52*, *MYB83*, and *MYB63*) of the transcriptional regulation network of secondary wall formation (Figure 9; Zhong *et al.* 2008, 2011). These results also demonstrated that miRNA858s participate in multiple biological processes by regulating different target genes among different plant species.

*HB-8* and *HB-15* play potential roles in xylem formation and are regulated by only one miRNA, miR166, in *Acacia mangium* (Ong and Wickneswari 2012). However, we observed that, when *HB-8* (Potri.018G045100) and *HB-15* (Potri.001G188800 and Potri.003G050100) participated in the biological processes of the late phase of wood formation of poplar (Figure 9), such as cell wall biogenesis, organization and macromolecule metabolic process, they were regulated by not only the miR166 family but also the miR165-y and miR5168-y (Figure 9). These results demonstrated different miRNA regulatory mechanisms of *HB-8* and *HB-15* involved in wood formation among different plant species. In a previous study, miR397 functions in the lignification of wood formation by regulating 29 *LACs* of the *LAC* family in poplar (Lu *et al.* 2013). We found that miR397s, including ptc-miR397a and miR397-x, were involved in the lignin catabolic, phenylpropanoid biosynthetic and metabolic processes of the late phase of wood formation only by regulating two target genes, *LAC7* (Potri.016G107500, Potri.006G094100) and *LAC11* (Potri.009G102700) (Figure 9). The difference in the number of target genes of miR397 might be caused by different miRNA identification methods or research material differences between the present and previous studies of poplar.

### Functions of the first identified miRNAs participate in diverse phases of wood formation

In addition to the above-described miRNAs, we also identified multiple miRNAs that are have not been reported to be related to wood formation. For example, we found that miR9776-x, with high expression level in TS and SS, participated in the beginning phase of wood formation by significantly alleviating the inhibitory effects on the target *TF CYCA3; 4* (Potri.014G021100), *CTL1* (Potri.014G146600), *PHH1* (Potri.008G166600) and *AFH1* (Potri.017G009900), which involved in meristem growth, cell cycle and growth processes (Figure 9). Ptc-miR390a might play roles in the beginning phase of wood formation by significantly alleviating the inhibitory effects on target structural genes *BAM3* (Potri.001G073600), *SARK* (Potri.006G179400), and *BRL2* (Potri.010G101100) (Figure 9), which related to meristem initiation, growth, maintenance, and cell growth processes. MiRNAs have function in PS also include miR5054-y, novel-m1442-3p, and ptc-miR394a/b-5p, which target *AESP* (Potri.003G021700), *HSL1* (Potri.017G016600) and *EMB2780* (Potri.005G065800), respectively (Figure 9), which involved in meristem initiation, growth, maintenance, structural organization, and cell cycle, cell differentiation, and cell growth processes. Target *AMPD* (Potri.016G119300) with high expression level in PS and TS, which participation in cell cycle, differentiation, division, and meristem structural organization multiple processes, was regulated by miR2662-y. They may play the key roles in the primary and middle stages of wood formation.

Moreover, miR6425-x was found to be involved in the late phase of wood formation by significantly alleviating the inhibitory effects on the target *PRR1* (Potri.001G133300) (Figure 9), which participation in xylan biosynthetic and metabolic processes. Of the remarkable GO terms for targets of DE-miRNAs, phenylpropanoid biosynthetic and metabolic process, and lignin metabolic process was the represented term, among which novel-m0260-5p and *C4H* (Potri.018G146100) was the key. This target has a highest expression level in SS, suggested novel-m0260-5p regulates lignin or phenylpropanoid biosynthetic and metabolic in the late phase of wood formation (Figure 9). In addition to the previously identified miRNAs, miR397s (Lu *et al.* 2013), of *LACs*, we also identified a new regulatory miRNA of *LAC*, novel-m1190-3p (Figure 9), which targeted *LAC2* (Potri.009G156800, Potri.009G156600) in SS; thus, it functioned in the late phase of wood formation.

Then, we find target *BEN1* (Potri.002G148000) with the highest expression level in SS, which participation in cellular metabolic process, was regulated by novel-m1395-5p and novel-m0738-5p. In previous study, *BEN1* was reported to play a key role in PCD, suggest novel-m1395-5p and novel-m0738-5p may indirect regulated the PCD process in the late phase of wood formation.

## Conclusions

Our study identified a battery of miRNAs including both known and many novel ones involved in diverse phases of wood formation varying from predominantly primary to secondary growth, and then interactively analyzed the miRNAs and mRNA expression profiles across diverse developmental stems of *P. trichocarpa*, shedding some light on many dynamic and diversified aspects, and details of miRNA regulation during vegetative growth to secondary wood formation. Our results demonstrated that miRNAs not only regulate some important *TFs* but also structural genes directly and indirectly involved in diverse processes of wood formation, suggesting that further studies of wood formation should also focus on miRNAs in order to obtain a holistic picture of the underlying molecular regulatory mechanisms. There are still a number of miRNAs with novel functions that need to be characterized. To our knowledge, this study is the first systematic investigation of miRNAs and their targets involved in diverse phases of wood formation using integrated analysis of miRNA and mRNA expression profiles. The copious information and data provided here will advance the roles of miRNAs and their regulatory functions in diverse phases of wood formation in tree species.

Overall, we finally identified 34, 6, and 76 miRNA-CTG pairs (Includes every member of shown miRNA family from Figure 9) from PS, TS, and SS, respectively, where miRNA target genes either regulate or are involved in cell division and differentiation, cell wall modification, SCW biosynthesis, lignification, and PCD processes.
